# Mechanical Properties of the Interfacial Bond between Asphalt-Binder and Aggregates under Different Aging Conditions

**DOI:** 10.3390/ma14051221

**Published:** 2021-03-05

**Authors:** Xiaorui Zhang, Juntian Wang, Xinxing Zhou, Zhuqiu Zhang, Xiaobing Chen

**Affiliations:** 1 School of Transportation, Southeast University, Nanjing 211189, China; 220183082@seu.edu.cn; 2 Key Laboratory of Highway Construction and Maintenance Technology in Loess Region, Shanxi Transportation Technology Research & Development Co., Ltd., Taiyuan 030032, China; zxx09432338@whut.edu.cn; 3 State Key Laboratory of Silicate Materials for Architectures, Wuhan University of Technology, Wuhan 430070, China; 4 Nanjing Design Branch, Pan-China Group, Nanjing 210019, China; zhangzhuqiu@suyi-group.com

**Keywords:** mechanical property, aggregate, tensile strength, elastic modulus, interfacial recovery energy, free volume

## Abstract

Aging has a detrimental impact on the interfacial interaction and bonding between asphalt-binder and aggregates, which influence ultimately on the performance of asphalt mixtures and pavements. Evaluation of the mechanical properties of the interface between the asphalt-binder and aggregates has thus become a hot research topic, particularly as a function of aging. In this study, the interfacial tensile strength, compressive strength, elastic modulus, and interfacial recovery energy were measured and quantified using molecular dynamic simulation. Whilst the free volume of the asphalt mixtures exhibited sensitivity to aging, the interfacial tensile strength decreased with an increase in the degree of aging. In general, the mechanical properties of the asphalt-binder-aggregate interface were found to be significantly dependent on the aggregate type. Furthermore, the study results indicated that interfacial recovery energy is a key characteristic property for characterizing the interfacial adhesive force within asphalt mixtures. Overall, the study of mechanical properties of the asphalt-binder and aggregate interface, as presented in this paper, contributes to quantifying the adhesive properties and improving the performance of asphalt mixtures.

## 1. Introduction

One of the common concerns in asphalt pavements is the aging effects of the asphalt-binder due to environmental exposure. Aging leads to changes in the chemical composition of the asphalt-binder and ultimately its mechanical properties, thus affecting the overall performance of the asphalt pavement [1,2]. The performance and service life of an asphalt pavement are closely related to the quality of the asphalt mixture. An asphalt mixture is mainly composed of reinforced, matrix, and interfacial phases, respectively. The reinforced phase consists of aggregate and mineral fillers. The matrix phase mainly constitutes the asphalt-binder whilst the interfacial phase comprises the interaction between the reinforced and matrix phases [3].

The reinforced phase, mostly aggregates, constitutes the skeleton and bears most of all the vertical loads. The shear and tensile stress caused by vehicle loading are mainly borne by the asphalt-binder matrix. The interface (i.e., interfacial bonding) is the bridge and link between the asphalt-binder and aggregate in the asphalt mixture. The mechanical properties of the asphalt mixture are mainly related to the adhesion properties of the asphalt-binder-aggregate interface [4]. However, due to the relatively complex material composition and low strength, this interfacial interaction is more likely to be damaged and cracked under the influence of external loading and environment [5].

With aging of the asphalt-binder, the chemical composition and physical properties of the asphalt-binder will change, ultimately affecting the adhesion of the asphalt-binder-aggregate interface and mechanical properties of the asphalt mixture [6]. That is, the adhesive strength of the interfacial bonding between the asphalt-binder and aggregate largely determines the overall stability of the asphalt mixture. Therefore, understanding and optimizing the asphalt-binder-aggregate interfacial bonding is critical towards maximizing the performance of asphalt mixtures.

### 1.1. Literature Review and Study Motivation

In general, the interaction between the asphalt-binder and aggregate interface is the decisive factor in the generation of the asphalt mixture strength and has a direct impact on the overall performance of the asphalt mixture and corresponding pavement structure [7]. The study of the interfacial mechanics between the asphalt-binder and aggregate, exploration of the interfacial failure mechanism, and the asphalt-binder-aggregate interaction ability is of great significance for the design, performance enhancement, and maintenance of asphalt pavements [8]. Tan et al. [9] successfully used the K. Ziegel-B interaction parameter as an evaluation index to characterize the asphalt-binder-aggregate filler interactions and used the dynamic shear rheometer (DSR) to quantify the rheological properties of four types of polymer powders using two asphalt-binders. By using variance analysis, various factors affecting the asphalt-binder-aggregate filler interactions were comparatively analyzed. The study showed that the factors contributing to the interaction sequence from high to low were the aggregate particle size, silica content, and filler concentration, respectively.

Rodrigues et al. [10] used the VIC-3D observation system and a computational software to observe the whole process of the semicircular bending test of an asphalt mixture and computationally modelled the strain evolution. The study evaluated the development characteristics and mechanical properties of the asphalt-binder-aggregate interface during crack initiation and development for two asphalt mixtures from a mesoscopic scale perspective. Fracture energy (FE) and fracture toughness (Jc) were successfully used to evaluate the fracture performance of the asphalt mixture. The cohesion model principle was used for modeling the laboratory specimens and thereafter, the viscoelastic parameters (namely complex shear modulus, phase angle, rutting factor, etc.) were derived using empirical formulae [11]. The failure process of the asphalt-binder-aggregate interface in the asphalt mixture was simulated and modeled using the ABAQUS finite element software [12]. The results showed that the numerical simulations and modeling were similar to those of the laboratory test.

Xu [13] used molecular dynamics (MD) to study the mechanical behavior of asphalt-binder-aggregate interface. The atomic model of the asphalt-binder-aggregate interface was established using the MD model. In the study, the adhesion and moisture damage of the asphalt-binder-aggregate interface were comparatively studied. The asphalt-binder chemical composition has a direct impact on the asphalt-binder performance and the asphalt-binder-aggregate interface including the resultant asphalt mixture and pavement structure. From the study, it was found that the MD simulation results were in good agreement with the experimental data and observation results. The MD simulations provided the deformation and failure mechanisms of the asphalt-binder-aggregate interface at an atomic scale. Overall, the study indicated that the basic chemical physics and mechanics of the asphalt-binders can be studied using MD simulations and numerical modeling.

Qiu et al. [14] studied the shear failure characteristics of the asphalt-binder-aggregate interface by researching the shear strength and continuous shear failure process of the asphalt-binder-aggregate interface. Firstly, the influence of the surface roughness, asphalt-binder aging, loading rate, and aggregate type on interfacial shear strength was studied using an oblique shear test. Secondly, the load-displacement (L-D) response curve of the continuous failure characteristic of the interface of the concrete structure was evaluated through the direct shear test. Finally, grey relational analysis (GRA) was used to identify and establish some correlations between the applied test conditions and the parameters of the interfacial bond-slip model. The results showed that an increase in the surface roughness and loading rate can improve the shear strength of the interfacial bonding. The study findings also indicated that aging of the asphalt-binder can also improve the shear strength of the interface, however, it is also detrimental to the shear strength of the interfacial bond.

Guo et al. [15] studied the viscoelastic parameters of different asphalt mixtures using the dynamic mechanical analysis (DMA) method and computed the interfacial interaction parameters of the asphalt-binder and filler according to the viscoelastic parameters. Thereafter, relevant theoretical models were used to compare the interaction parameters. The corresponding results showed that the sensitivity of the C-value based on the Palierne theoretical model to the volume fraction of packing was the least as compared with Luis Ibrarra-A and K. Ziegel-B model formulations.

Su et al. [6] adopted two self-developed testers, namely the aggregate contact-shear and interface shear-slip testers, to measure the parameters of the particle contact and interface shear-slip for mesoscopic level analysis. The results showed that the interfacial interaction is mainly caused by the coupling effect of the particle contact and the asphalt-binder bonding lubrication.

Yang et al. [16] pointed out nanolayered silicate could improve the high-temperature rheological properties and inhibited the molecular movement of asphalt through the internal network structure formed by nanoparticles in asphalt. Yao et al. [17] investigated the functional group changes of nano-modified asphalt binders containing polymer modified nanoclay, nonmodified nanoclay, and nanosilica after short-term RTFO aging and long-term PAV aging using Fourier Transform Infrared Spectroscopy (FTIR) test. Wang et al. [18] found carbon nanomaterials could improve the thermodynamic parameters of modified asphalt after short-term RTFO aging and reduce the these parameters after long-term UV aging. Xu et al. [19] found nano-ZnO could significantly improve fatigue-related rheological properties and the anti-UV aging ability of asphalt. Zhang et al. [20] found the additions of 1% OEVMT and 3% nano-ZnO could lower viscosity aging index, softening-point increment, phase angle aging index, and complex modulus aging index, indicating the best anti-aging properties.

Qian et al. [21] found the anti-ultraviolet aging performance of asphalt was obviously improved because of the barrier effect of nano-SiO_2_ and carbon black in rubber powder. Cheraghian et al. [22] found the clay and fumed silica nanoparticles could reduce the index of carbonyl and oxidation degree, thus improving aging resistance to ultraviolet (UV) radiation. Bhat et al. [23] found nano-Al_2_O_3_ could improve Superpave rutting parameter and prolong the fatigue life of modified asphalt. Al-Omaria et al. [24] found the additions of crumb tire rubber and nanosilica increased the resistance to rutting, which is manifested by the increasing rutting parameter (G*/sinδ). Motamedi et al. [25] found nanosilica and synthesized polyurethane improved fatigue performances of the asphalt binder and mastic through time sweep (TS) and linear amplitude sweep (LAS) tests. Liu et al. [26] found the addition of oxidized crumb rubber and nano-SiO_2_ could significantly improve the rheological properties of asphalt binder at high temperatures.

### 1.2. Study Objectives and Scope of Work

To better understand the mechanical properties of the interfacial interaction between the asphalt-binder and aggregate, the tension strength, compressive strength, elastic modulus, interfacial energy, interfacial bonding force, and free volumes of the asphalt-binder and Al_2_O_3_/SiO_2_/CaO minerals under different aging conditions were evaluated and quantified using molecular dynamic simulations. The mechanisms of interfacial interaction between the aged asphalt-binder and the aggregates were also investigated and are discussed in this study.

In the subsequent sections of the paper, the study methodology, work plans, and laboratory experimentation are discussed, followed by molecular modeling and dynamic simulations of the asphalt-binder-aggregate interfacial interactions. The results are then presented along with corresponding analysis and syntheses. The interfacial interaction between the aged asphalt binder and aggregates were also investigated.

## 2. Study Methodology and Materials

To accomplish the study objectives, the research method-ology and scope of work incorporated executing the following key tasks, namely: (a) Material sourcing and procurement; (b) sample preparation; (c) molecular modeling and dynamic simulations; (d) laboratory experimentation and testing; and (e) data analysis and synthesis thereof. Whilst the rest of the work tasks are discussed in the subsequent texts, the materials and sample preparation are described in this section of the paper.

### 2.1. Asphalt-Binder

The base asphalt-binder used was a 70# asphalt-binder sourced from Panjin Northern Asphalt Co. LTD. (Panjin, China), with a penetration of 66 dmm (0.1 mm). Its softening point (ring and ball temperature of 45 °C) was measured according to the ASTM D36/D36M-14 [27] test procedure. The ductility, measured based on the ASTM D113-17 [28] test procedure, at 15 °C was 128 cm.

### 2.2. Aggregate Mineral

**Figure 1:**
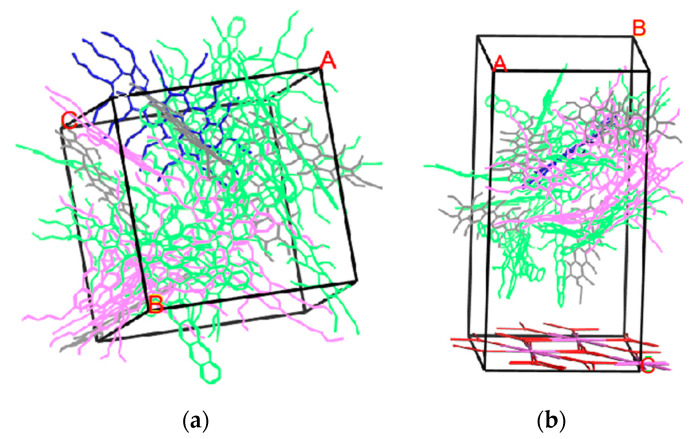

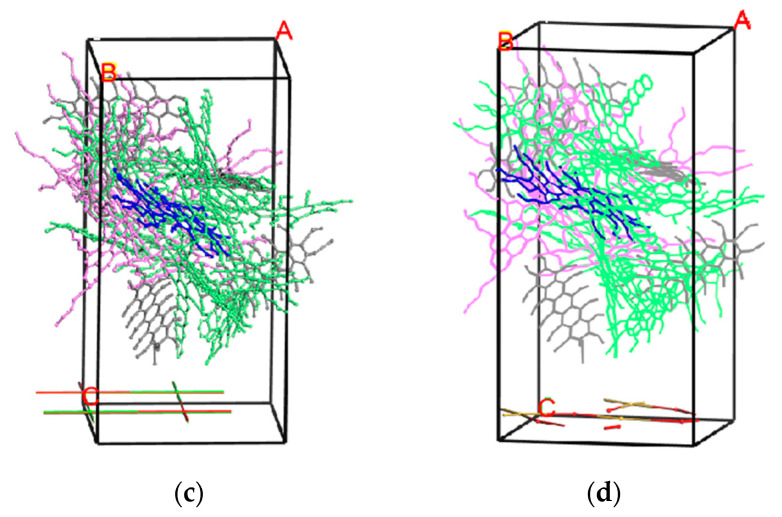
The interfacial models of the asphalt-binder-mineral bond: (**a**) asphalt binder (blue represents aromatics; grey represents saturates; green are asphaltenes; and purple are resins); (**b**). asphalt-binder-Al_2_O_3_; (**c**) asphalt-binder- SiO_2_; (**d**) asphalt-binder- CaO.

In this study, basalt, aluminous rock, and limestone were represented using Al_2_O_3_/SiO_2_/CaO minerals. To measure the tensile strength between the asphalt-binder and aggregate interaction, the basalt, aluminous rock, and limestone were processed into cuboid blocks with a smooth surface. The interfacial models showed in Figure 1 (A, B and C are coordinates of x, y and z in simulation software, respectively).

## 3. Molecular Dynamic Modeling

Molecular modeling and dynamic simulations included quantifying the interfacial mechanical properties, interfacial energy, and adhesive work of the asphalt-binder-aggregate (mineral) bond interaction. For the asphalt, we selected a four components model, and for the detailed molecular components, refer to Zhou [29]. For the mineral component we selected three different oxides (Al_2_O_3_/SiO_2_/CaO) from the models library of Materials Studio software 8.0. The asphalt model was built first, then the asphalt binder-aggregate model was built using a layer model. The layer model of asphalt binder-aggregate model was treated by geometry optimization. Additionally, the NVT and NPT ensembles were used to calculate the detailed parameters with the total times of 100 ps. The simulated density and energy values were verified by experiments first. If, the simulated data can be identical with experiments, only the data of molecular dynamic simulation can be used. These model simulations and their corresponding mathematical computations are discussed in the subsequent texts.

### 3.1. Mechanical Modeling and Parametric Computations

The molecular dynamics method [30] was used to simulate and compute the mechanical parameters including the interfacial tensile strength, compression strength, and elastic modulus. The strain was considered as a parametric constant during both the tension and compression processes, respectively. The quantitative value of the tensile and compression strength were obtained from the elastic region of stress-strain response curve, with the tensile strength, σ, computed using Equation (1).

(1) $$\sigma =\frac{F_{\max}}{S}$$

where *F*_max_ is the maximum stress of the elastic region for the stress-strain response curve, *S* is the original sectional (sample) area. The compression strength, *P*, was calculated by Equation (2) as follows:

(2) $$P=\frac{F}{S}$$

where *F* is the maximum fracture stress, *S* is the original sectional (sample) area. For elastic materials, Hooke’s Law [31] can be used to describe its elastic properties, as illustrated in Equation (3):

(3) $$\sigma_{i}=C_{\mathrm{ij}}\varepsilon_{j}$$

In Equation (3), *i*, *j* = 1, 2, 3, etc., whereas σ_i_ and ε_j_ are the stress and strain components, respectively, and *C*_ij_ is the stiffness matrix in the six axial directions. Assuming that the maximum allowable stress is 0.03 MPa, the stress can be estimated Equation (4) as follows:

(4) $$\sigma_{\mathrm{ij}}=-\frac{1}{V}\underset{k}{\sum }\left[m^{k}\left(u_{i}^{k}u_{j}^{k}\right)+\frac{1}{2}\underset{i\neq k}{\sum }\left(r_{i}^{\mathrm{kl}}\right)f_{j}^{\mathrm{lk}}\right]$$

In Equation (4), the Lambda coefficients λ and μ, which are related to the elastic constants, can be computed using Equation (5) through to Equation (7):

(5) $$\lambda =\frac{1}{6}\left(C_{12}+C_{13}+C_{21}+C_{23}+C_{31}+C_{32}\right)\approx \frac{1}{3}\left(C_{12}+C_{23}+C_{13}\right)$$

(6) $$\mu =\frac{1}{3}\left(C_{44}+C_{55}+C_{66}\right)$$

(7) $$\lambda +2\mu =\frac{1}{3}\left(C_{11}+C_{22}+C_{33}\right)$$

The elastic modulus is a physical quantity that characterizes the tensile or compressive resistance of a material within the elastic limit [32,33,34]. Young’s modulus (*K*) can be calculated from the elastic constants, namely the Lambda coefficients λ and μ expressed in Equations (5) and (6). The specific formulation for computing Young’s modulus, *K*, is illustrated in Equation (8).

(8) $$K=\frac{\mu \left(3\lambda +2\mu \right)}{\lambda +\mu }$$

### 3.2. Interfacial Energy Simulation and Computations

The Kinetic equation of aggregation and growth of the second phase particles can be used to determine the phase boundary energy between the matrix phase and the second phase particles [35]. The corresponding mathematical formulation is expressed in Equation (9) below:

(9) $$r^{3}-r_{0}^{3}=\frac{8}{9}\frac{\gamma DCV^{2}}{k_{B}T}t$$

In Equation (9), γ is the phase boundary energy, *r*_0_ is the average radius of the second phase particles before they start to grow, *r* is the average radius of the particles at temperature *T* and time *t*, *D* is the diffusion coefficient of the solute in the matrix phase, and *C* is the temperature *T* when there is equilibrium solubility for the solute in the matrix phase. Parameter *V* is the atomic volume of the second phase, and *k*_B_ is the Boltzmann constant [36].

### 3.3. Adhesive Work Modeling and Computations

This study mainly considered the tensile stress perpendicular to the interface and solved the interfacial adhesion strength by recording the stress-strain change of the interface under tensile stress loading. The adhesion work was simulated and computed using the mathematical model shown in Equation (10):

(10) $$W_{a}=\frac{\left(E_{t}-\left(E_{a}+E_{b}\right)\right)}{A}$$

In Equation (10), *E*_a_ represents the potential energy of separation of the asphalt-binder; *E*_b_ represents the potential energy of separation of the aggregate; *E*_t_ represents the total potential energy of the asphalt-binder and aggregate; *A* represents the contact area of the asphalt-binder and aggregate; and *W*_a_ represents the adhesion work or the work of adhesion, i.e., work done due to the adhesion between the asphalt-binder and aggregate particles.

## 4. Nano-Indentation Testing

**Figure 2:**
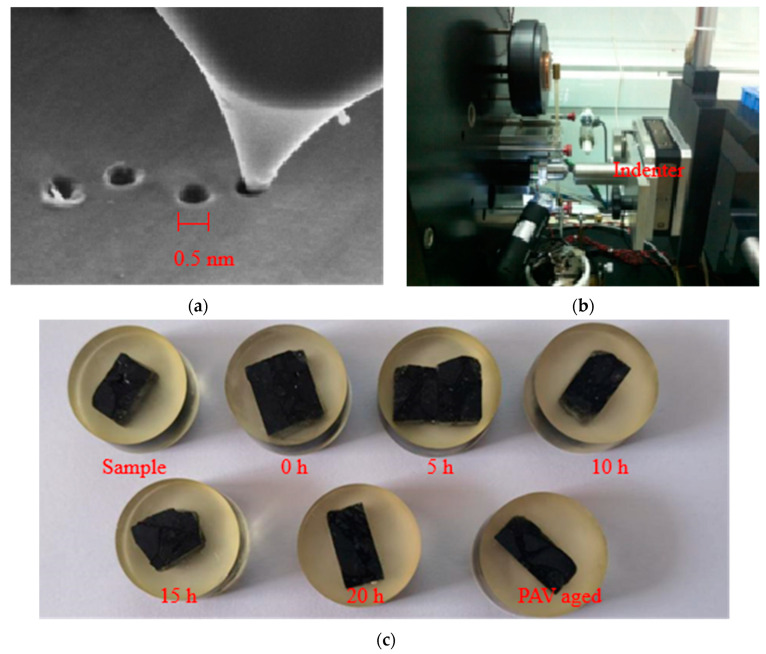
Photographical illustration of the nano-indentation test procedure: (**a**) the scale of experiment; (**b**) nano-indentation test equipment; (**c**) test samples under different aged time.

Nano-indentation, also called instrumented indentation, is a microscopic indentation test [37]. This means that a diamond indentation is used to touch the flat surface of a sample material at a nanoscale. A photographical illustration of the indentation test, along with the test setup, is shown in Figure 2a,b, respectively.

Figure 2a is a photographical illustration of the process of indenting asphalt materials with a diamond tip. Figure 2b is the parts of the test equipment, including indenter head and displacement sensor, asphalt sample, and sample thermocouples, etc. Figure 2c is the asphalt sample for nano-indentation testing.

When the indentation is pressed into the sample surface, the load and depth of the indentation are recorded to generate the load-displacement response curve. In this study, the FemtoTools NanoIndentor was used for the indentation measurements. The indentation displacement ranged from 0.05 nm to 21 mm. In this study, the indentation test was performed on asphalt-binder-aggregate mixture samples as per ASTM E2546-15 [38]. The adopted samples preparation procedure was as follows:

- Cut the asphalt mixture (i.e., a mixed blend of asphalt-binder and aggregates) into small square sample pieces of about 1.5 cm length to cover the test area;
- mix, stir, and pour epoxy resin (about 25 g) together with the small square sample pieces of the mixture into cylindrical molds of 2.5 cm diameter;
- when curing of epoxy resin adhesive was completed (i.e., after 3 h), the sample should be polished until the surface is smooth—see Figure 2c for some test samples.

## 5. Results and Discussion

The results of the asphalt-binder-aggregate bonding interaction are presented, analyzed, and discussed in this section. These results include the mechanical properties, interfacial energy, and adhesion work from molecular modeling as well as the mechanical responses from the nano-indentation testing.

### 5.1. Mechanical Property Results

**Figure 3:**
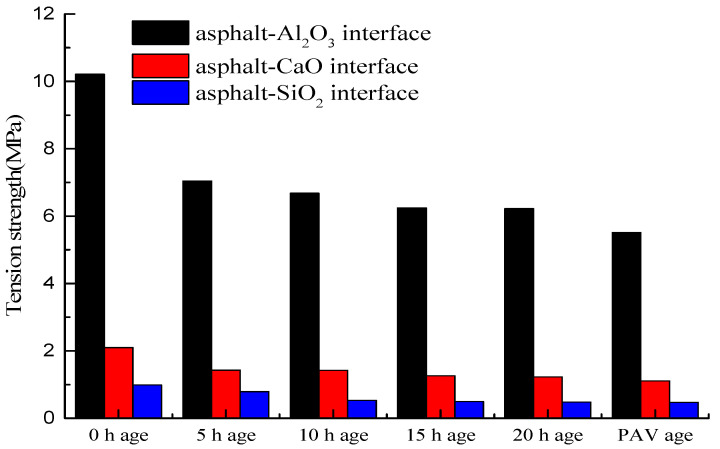
Tensile strength of the asphalt-binder aggregates interface. (PAV = Pressure Aging Vessel).

In this study, the mechanical properties of the interface between the asphalt-binder and aggregates mainly included the interfacial tensile strength, compressive strength, and elastic modulus, which were quantified to better understand the interaction between the asphalt-binder and aggregates. As shown in Figure 3, the tensile strength of asphalt-binder-aluminous rock (Al_2_O_3_) interface is significantly greater than that of the asphalt-binder-basalt (SiO_2_) interface and asphalt-binder limestone (CaO) interface, respectively. The reason is that the Al_2_O_3_ could form the covalent bond with asphalt components, while the CaO could not. Moreover, atoms have overflowed from the layer of CaO. This suggests better affinity and bonding for the asphalt-binder and the aluminous rock aggregates.

From Figure 3, it is also evident that the tensile strength of the asphalt-basalt interface is relatively higher than that of the asphalt-limestone interface. The stress-strain response curves showed that there were elastic and viscosity (plastic yielding) areas within the asphalt-binder-mineral matrix during the tension process. These elastic regions are the primary key to generating the tensile strength. The tensile strengths of the different aged asphalt-binder-mineral interface results showed that there exists different tensile strengths during the different aging conditions of the asphalt-binder-mineral interface. With an increase in the degree of aging, the tensile strength of the asphalt-binder-minerals interface is decreased. Furthermore, Figure 3 shows a general decline in the tensile strength with an increase in the aging time. Overall, the results indicated that aggregate (mineral) type and aging time could significantly affect the tensile strength of the asphalt-binder-aggregate (mineral) interface. Furthermore, the effects of the pressure aging vessel (PAV) on tensile strength was observed to be more pronounced than that of 0–20 h short-term aging.

**Figure 4:**
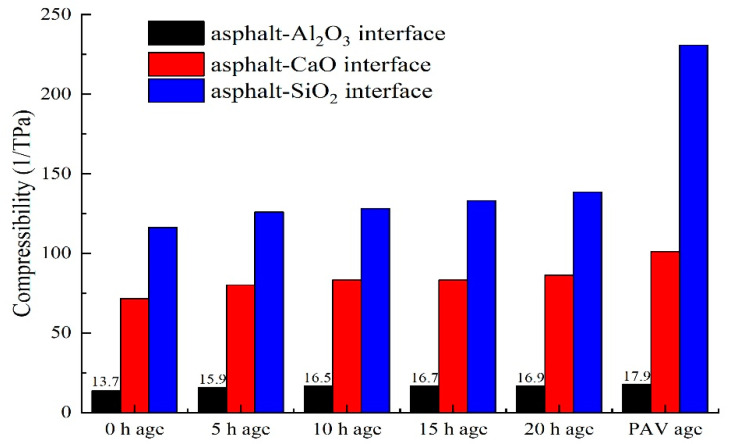
Compressibility of the asphalt-binder-aggregate mineral interface.

As shown in Figure 4, compressibility was used to evaluate the compressive strength of the asphalt-binder-aggregate mineral interface. As can be seen in the figure, the compressibility of the asphalt-binder-aggregate mineral interface is different for different aggregate types, aging time, and aging type. Overall, the compressibility of the asphalt-binder-aggregate (mineral) interface increased with aging time and aging type.

As can be seen from Figure 4, the compressibility of the asphalt-binder-aluminous (Al_2_O_3_) interface is smaller than that of the asphalt-binder-basalt and asphalt-binder-limestone interfaces, respectively, which is basically like the tensile strength results show previously in Figure 3. That is low compressibility indicates high compressive strength and vice versa. Overall, the largest compressibility (i.e., lowest compressive strength) was registered for the asphalt-binder-limestone interface and the least for the asphalt-binder-aluminous (Al_2_O_3_) interface, i.e., highest compressive strength. In general, the compressibility of the asphalt-binder-aggregate (mineral) interface increased with aging time and the degree of aging, with the PAV aged asphalt-binder-aggregate (mineral) interface exhibiting the highest compressibility, i.e., lowest compressive strength. In general, the results in Figure 4 indicated that both PAV and short-term aging could potentially improve the compressibility of the asphalt-binder-aggregate (mineral) interface. Similarly, aggregate (mineral) type, aging time, and aging type were also observed to have an effect on the compressive properties of the asphalt-binder-aggregate (mineral) interface. For application in asphalt mixtures, attention should thus be given to the effects of PAV aging and aggregate types on the compressive properties of the asphalt-binder-aggregate (mineral) interface and the corresponding asphalt mixtures.

**Figure 5:**
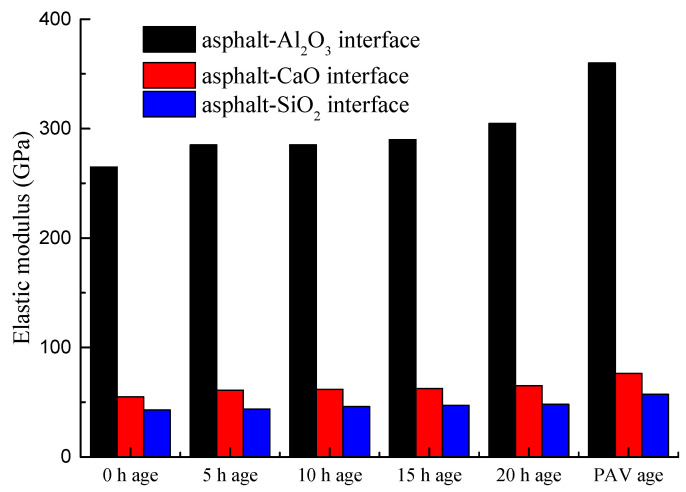
The elastic modulus of asphalt-binder aggregate mineral interface.

As shown in Figure 5, the elastic modulus of the asphalt-binder-aggregate (mineral) interface exhibited a different response trend to that of the tensile strength and compressibility results in Figure 3 and Figure 4, respectively. That is the change in the elastic modulus as a function of aging time was not significant. However, the elastic modulus of the asphalt-binder-aggregate (mineral) interface increased with an increase in the degree of aging. For example, the elastic modulus of the PAV aged asphalt-binder-aggregate (mineral) interface is larger than that of the 0–20 h aged asphalt-binder-aggregate (mineral) interface, and so, is that of the 20 h over the 15 h short-term aged moduli results. Nonetheless, it is apparent from Figure 5 that different aggregate types exhibited different respond trends with respect to the elastic modulus analysis.

In general, the elastic modulus of the asphalt-binder-aluminous (Al_2_O_3_) interface was observed (Figure 5) to be larger than that of the asphalt-binder-basalt and asphalt-limestone interfaces, respectively. As theoretically known, the elastic modulus of the basalt rock aggregates is generally higher than that of limestone rock aggregates 38, which is consistent with the bar-chart results in Figure 5 when comparing CaO to SiO_2_. Overall, the results in Figure 5 indicated that the elastic modulus of the asphalt-binder-aggregate (mineral) interface is a function of the aggregate type and that the aggregate (mineral) characteristic properties are instrumental to quantifying the elastic modulus of the asphalt-binder-aggregate (mineral) interface.

### 5.2. Interfacial Energy Results

**Figure 6:**
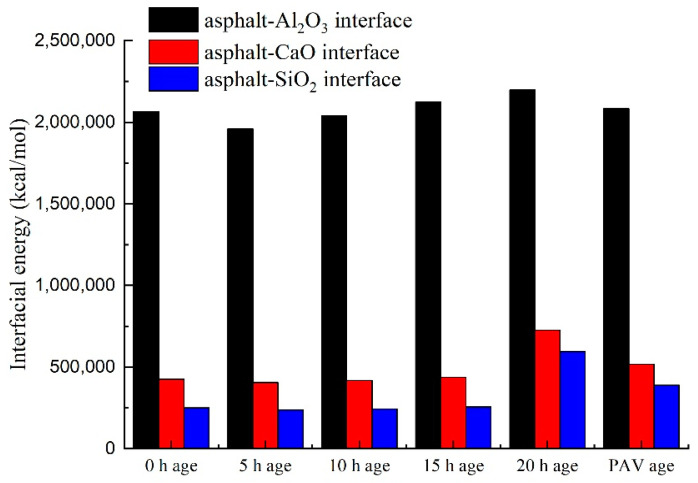
Interfacial energy of the asphalt-binder-aggregate mineral interface.

To evaluate the interfacial properties of the asphalt-binder-aggregate (mineral) interface, the interfacial energy was simulated and quantified as a function of different aging times, aging types, and aggregate types. Theoretically, the lower the interfacial energy in magnitude, the poor the adhesive properties of asphalt-binder-aggregate (mineral) interface, and vice versa. The interfacial energy results for the materials evaluated in this study are shown in Figure 6.

As shown in Figure 6, the interfacial energy of the asphalt-binder-aggregate (mineral) interface did not change significantly as a function of aging, but was considerably different for different aggregate (mineral) types. In the figure, the interfacial energy is relatively higher at 0 h aging, decreases slightly for 5 h aging, and then increases progressively through to 20 h short-term aging, and thereafter declines slightly again for PAV aging. In general, the results in Figure 6 suggests that aggregate type (e.g., Al_2_O_3_) was more impactful on the interfacial energy of the asphalt-binder-aggregate (mineral) interface than aging. That is oxidative aging with time can detrimentally promote stripping of the asphalt-binder from the aggregates (minerals) whilst the use of aggregates (minerals) such as Al_2_O_3_ can potentially aid to mitigate stripping. Overall, it can be concluded from Figure 6 that aggregates rich in the Al_2_O_3_ mineral had good adhesion with the asphalt-binder and registered a high interfacial energy with high potential for stripping mitigation.

### 5.3. Adhesive Work Results

**Figure 7:**
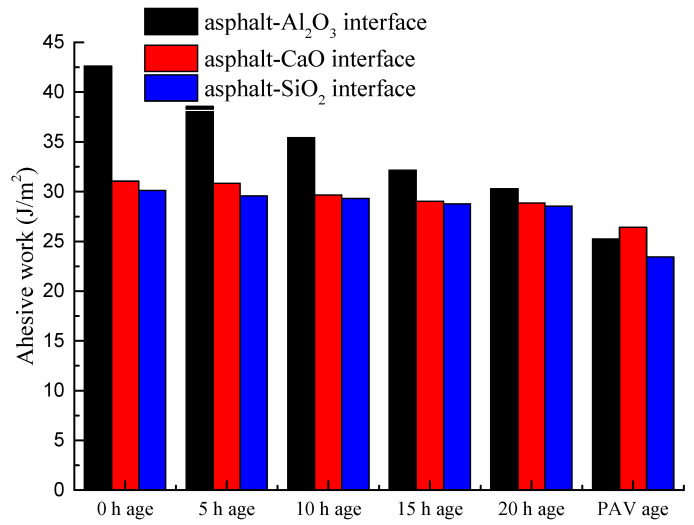
Adhesive work of asphalt-binder-aggregate mineral interface.

In this study, the adhesive work concept was used to model and better understand the interfacial properties of the asphalt mixtures, particularly with respect to adhesive bonding. The higher the adhesive work is, the better the adhesive properties and bonding of the asphalt-binder-aggregate (mineral) interface are, and vice versa. With the exception of PAV aging, Figure 7 shows that the adhesive work of the asphalt-binder-aluminous rock (Al_2_O_3_) interface is the highest followed by CaO and lastly, SiO_2_.

As shown in Figure 7, the adhesive work generally exhibited a declining trend as a function of aging, with the lowest numerical values registered for PAV aging. Thus, it is evident from these results that aging is detrimental to the adhesive bonding of the asphalt-binder-aggregate (mineral) interface. Quite often, however, the novelty of how to investigate and adequately quantify the adhesion mechanisms of the asphalt-binder-aggregate (mineral) bonding interactions has been a research challenge. Nonetheless, some studies [30,31] have reported that the adhesion mechanisms of the asphalt-binder-aggregate (mineral) interface is mainly physical adsorption and the intermolecular forces, with the Van der Waals interactions being the main governing forces. Overall, these results substantiate that aggregate type, aging time, and degree of aging have a profound effect on the adhesion mechanism and adhesive properties of the asphalt-binder-aggregate (mineral) bonding interactions.

### 5.4. Nano-Indentation Test Results

**Figure 8:**
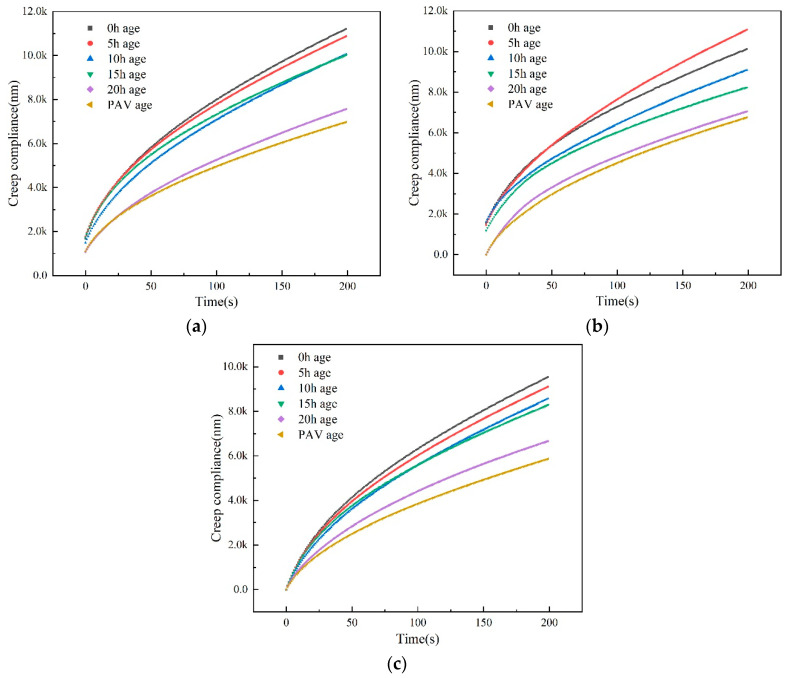
Nano-indentation mechanical response curves of different asphalt mixtures: (**a**) Basalt aggregates; (**b**) aluminous aggregates; (**c**) limestone aggregates.

To verify the dynamic modeling and simulation results, the micro-mechanical nano-indentation response-behavior of the asphalt mixtures (namely the asphalt-binder-aggregate mixture samples) were investigated and comparatively quantified as a function of aging. As shown in Figure 8, the creep deformation of the asphalt mixtures decreased with an increase in aging time, with the PAV aged samples registering the lowest creep deformation. That is the depth of indentation decreased as the aging time increased, with the least depth of indentation recorded for the PAV aged samples. As theoretically expected, the highest indentation was registered for the 0 and 5 h aged samples followed by the 10- and 15-h aged samples. As can be seen in the figure, the 20 h and PAV aged samples’ creep compliance response curves are relatively close to each other for all the aggregate (mineral) types.

However, the response curves in Figure 8 shows different depths of indentation for different aggregates, particularly the magnitude of limestone, which is the lowest at below 10,000 nm. Overall, these results substantiate that aggregate (mineral) type and aging have a profound effect on the micro-mechanical responses and indentation characteristics of the asphalt mixtures, namely the asphalt-binder-aggregate mixtures.

## 6. Conclusions and Recommendations

The mechanical properties of the interface between asphalt-binder (70#) and aggregates (namely basalt, aluminous rock, and limestone) under different aging conditions were investigated, and the following conclusions and recommendations were drawn:

- There are elastic and viscosity (plastic yielding) regions within the asphalt-binder-aggregate (mineral) interface during the tension process;
- the interfacial tensile strength varied as a function of aging and aggregate type. Thus, it was concluded that aggregate (mineral) type and aging time have a significant effect on the tensile strength of the asphalt-binder-aggregate interface;
- the interfacial energy of the asphalt-binder-aggregate bonding interaction exhibited dependency on aging time, degree of aging, and aggregate type. These findings suggested that aging with time can detrimentally promote stripping of the asphalt-binder from the aggregates whilst the use of aggregates with Al_2_O_3_ minerals can potentially aid to mitigate stripping;
- aggregate type, aging time, and degree of aging were found to have a profound effect on the adhesion mechanisms and adhesive properties of the asphalt-binder-aggregate bonding interaction
- similarly, the micro-mechanical responses and indentation characteristics of the asphalt mixtures were found to be sensitive to the aggregate type, and, also varied as a function of aging time.

Future follow-up studies should thus incorporate a wide array of materials (i.e., different asphalt-binders, aggregates, etc.) and moisture sensitivity tests along with field data correlations to further supplement the findings reported in this paper.

## Data Availability

The data presented in this study are available on request from the corresponding author.

## Abbreviations

DMA	Dynamic mechanical analysis
DSR	Dynamic shear rheometer
FE	Fracture energy
FTIR	Fourier Transform Infrared Spectroscopy
GRA	Grey relational analysis
Jc	Fracture toughness
LAS	Linear amplitude sweep
L-D	Load-displacement
MD	Molecular dynamics
OCR	Oxidized crumb rubber
OEVMT	Organic expanded vermiculite
PAV	Pressure aging vessel
RTFO	Rolling thin film oven
TS	Time sweep
UV	Ultraviolet
